# Nutrition mediates the relationship between number of teeth and sarcopenia: a pathway analysis

**DOI:** 10.1186/s12877-022-03350-7

**Published:** 2022-08-08

**Authors:** Xin Xia, Zhigang Xu, Fengjuan Hu, Lisha Hou, Gongchang Zhang, Xiaolei Liu

**Affiliations:** 1 National Clinical Research Center for Geriatrics, West China Hospital, Sichuan University, No. 37, Guo Xue Xiang Renmin Nan Lu, Chengdu, Sichuan China; 2 Geriatric Health Care and Medical Research Center, Sichuan University, Sichuan Province, Chengdu, China

**Keywords:** Sarcopenia, Number of teeth, Nutrition, Mediation

## Abstract

**Objectives:**

The relationship between the number of teeth and sarcopenia remains poorly investigated. Although nutrition plays an important role in maintaining bone and muscle health, the complex relationship between number of teeth and nutrition in the pathogenesis of sarcopenia remains to be elucidated.

**Methods:**

A large multi-ethnic sample of 4149 participants aged over 50 years old from West China Health and Aging Trend (WCHAT) study was analyzed. We examined the associations between number of teeth with nutritional status and sarcopenia, and the mediating role of nutrition in the association between number of teeth and sarcopenia. Sarcopenia was defined according to the Asian Working Group for Sarcopenia 2019. We assessed nutrition using Mini Nutrition Assessment-Short Form (MNA-SF) scale. Direct relationships between number of teeth, nutrition and sarcopenia were assessed using multiple linear regression. Mediation models and structural equation model (SEM) pathway analysis were used to test the mediating role of nutrition in the relationship between number of teeth and sarcopenia.

**Results:**

Of 4149 participants aged 50 years old or older, the prevalence of sarcopenia was 22.5, 9.0% for moderate sarcopenia, and 13.5% for severe sarcopenia, respectively. Regression analysis indicated a total association between number of teeth (β = − 0.327, 95% CI − 0.471 to − 0.237, *p* < 0.001) and sarcopenia. After adjusted MNA-SF scores, the association between number of teeth and sarcopenia was still significant (β = − 0.269, 95% CI − 0.364 to − 0.175, *p* < 0.001), indicating a partial mediation effect of nutrition. Mediation analysis verified nutrition partially mediate the associations between number of teeth and sarcopenia (indirect effect estimate = − 0.0272, bootstrap 95% CI − 0.0324 to − 0.0222; direct effect estimate = − 0.0899, bootstrap 95% CI − 0.1049 to − 0.0738). And this mediation effect was through impacting SMI (indirect effect estimate = − 0.0283, bootstrap 95% CI − 0.0336 to − 0.0232) and grip strength (indirect effect estimate = − 0.0067, bootstrap 95% CI − 0.0094 to − 0.0043). Structural equation model (SEM) framework pathway analysis confirmed the association between number of teeth, nutrition, and sarcopenia.

**Conclusions:**

Our findings indicated that sarcopenia was associated with number of teeth and poorer nutritional status, with nutrition partially mediating the association between number of teeth and sarcopenia. Our findings supported early nutritional assessment and intervention in oral health to mitigate the risk of sarcopenia.

## Introduction

The world is facing big challenges of an aging population. It was estimated that a large population of 703 million people were over 65 years old in 2019 and that this population would reach 1.5 billion by 2050 globally [1]. China is an aging country right now with a large elderly population. During the process of ageing, sarcopenia is an urgent problem that needs to be solved. Sarcopenia is characterized by the gradual loss of skeletal muscle mass and function with age and was established as a disease in ICD-10-CM (M62.84) [2]. The prevalence of sarcopenia varies widely among studies depending on the assessment method and population, with the prevalence of sarcopenia in the elderly community ranges from 9.9 to 40.4% [3, 4]. The mechanism of sarcopenia is complex and includes hormonal changes, nutritional deficiencies, chronic inflammation, neuromuscular function decline, and decreased physical activity [5].

Recently, oral health is attracting more and more attention. Assessment of oral health includes teeth number, chewing ability, articulatory and oral motor skill, tongue pressure, eating and swallowing ability [6]. These oral health problems could affect the intake of nutritious food and might lead to malnutrition, frailty or sarcopenia finally. Specifically, tooth loss is a common health problem among the elderly. The number of teeth was found be related with handgrip strength, skeletal muscle mass, or sarcopenia in some studies [7–9]. Besides, the number of teeth was significantly correlated with chewing function and dietary intake, causing malnutrition [10, 11], leading to muscle loss eventually. This suggested a potential association between number of teeth, nutrition, and sarcopenia. Therefore, in this study, we used mediation analysis to determine if nutrition status mediated the effects of teeth number on sarcopenia.

## Method

### Study design and data collection

**Fig. 1:**
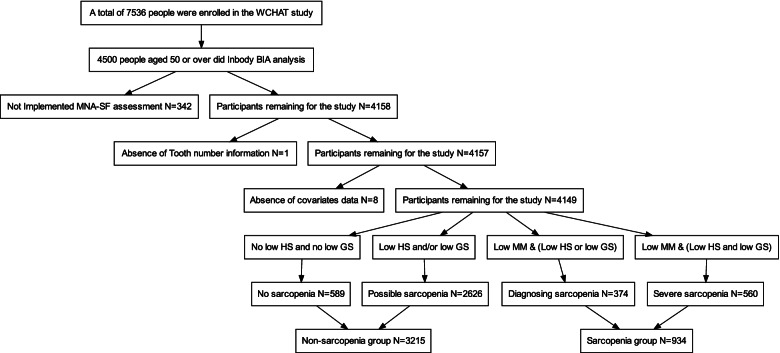
Flow chart of study participants. Initially, 7536 participants were enrolled and only 4500 participants did Bioelectric Impedance Analysis (BIA) analysis over 50 years old. Then we kept on excluding 342 subjects without nutrition assessment. Then 1 subject was excluded with missing information of teeth number. After that, 8 subjects were excluded without covariates data. Therefore, 4149 participants were analyzed in our study

We analyzed the baseline data from the West China Health and Aging Trend (WCHAT) study, which was approved by the Ethical Review Committee of West China Hospital (Committee reference number: 2017(445); Registration number: ChiCTR 1,800,018,895). Previous studies have published details of the questionnaires used to generate this data [12]. Participants aged over 50 years old were enrolled. Before the interview, participants were asked about their willingness to take part in the study and signed the informed consent. Initially, we enrolled 7536 participants. However, only 4500 participants over 50 years old did Bioelectric Impedance Analysis (BIA) analysis. Then 342 participants with missing nutrition information were excluded. Besides, 1 participant was excluded without the information of teeth number. After that, 8 participants were excluded without covariates data. At last, 4149 participants were analyzed in our study (Fig. 1).

We collected demographic characteristics including age, gender, ethnicity, educational levels, and marriage status. Lifestyle characteristics including tea drinking, alcohol drinking, and smoking were collected. Whether living alone and have false teeth were also asked. Participants self-reported medical history of chronic diseases including hypertension, osteoarticular disease, coronary heart disease, lung disease, diabetes, tumors, and others. Cognitive function was assessed using a 10-item Short Portable Mental Status Questionnaire (SPMSQ) [13]. Sleep quality was assessed using the Pittsburgh Sleep Quality Index (PSQI). And scores > 5 are considered as poor self-reported sleep quality. Depression and anxiety were evaluated using the 15-item Geriatric Depression Scale (GDS-15) [14] and Generalized Anxiety Disorder (GAD-7) [15] questionnaire, separately. And the scores ≥5 were considered as having depression or anxiety in the evaluation. Nutrition status was graded using the Mini Nutrition Assessment-Short Form (MNA-SF) scale. And MNA-SF scores from 0 ~ 7 indicated malnutrition status, 8 ~ 11 indicated malnutrition risk, 12 ~ 14 indicated good nutrition status [16].

### Sarcopenia evaluation

Sarcopenia was diagnosed with the Asian Working Group for Sarcopenia (AWGS) 2019 consensus criteria, defined as low relative appendicular skeletal muscle mass index (SMI) in the presence of either low handgrip strength or slow gait speed using updated cutoffs [17]. Bioelectric Impedance Analysis (BIA) was used to test muscle mass by the INbody770 body composition instrument. The cut-off value of the skeletal muscle mass index (SMI) was 7.0 kg/m [2] in male and 5.7 kg/m [2] in female [17]. Grip strength was measured with a grip dynamometer (EH101; Camry, Zhongshan, China). During the test of grip strength, participants was required to held the grip dynamometer with their dominant hand, keep their feet separated (shoulder-width apart), stand upright, with their arms drop naturally. The test should be repeated twice and the largest value was recorded. The cut-off value of grip strength was 26 kg in male and 18 kg in female. Gait speed was tested using an infrared sensor [18]. During the test, participants wore common shoes and was required to walk 6 m. The cut-off value of gait speed was 1.0 m/s.

### Statistical analysis

Statistical analysis was performed by using the R software (version 4.0.2). The characteristics of the population were showed as means±standard deviation (SD) or frequencies. Differences of the variables between the sarcopenia and non-sarcopenia group were analyzed. For continuous variables, one-way analysis of variance (ANOVA) was carried out. For categorical variables, chi squared test was carried out. Associations with a *p*-value of 0.1 or less in the univariate analysis were selected for the multiple regression analysis, which were used to estimate the odds ratio (OR) to identify associations between number of teeth, MNA-SF scores and sarcopenia after adjusting for potential confounders. A multi-categorical mediation model examined the potential mediating role of nutrition, using total MNA-SF score, in the relationship between number of teeth and sarcopenia. And a pathway analysis was shown in the SEM framework using a SEM package in R version 4.0.2 [19].

## Results

**Fig. 2:**
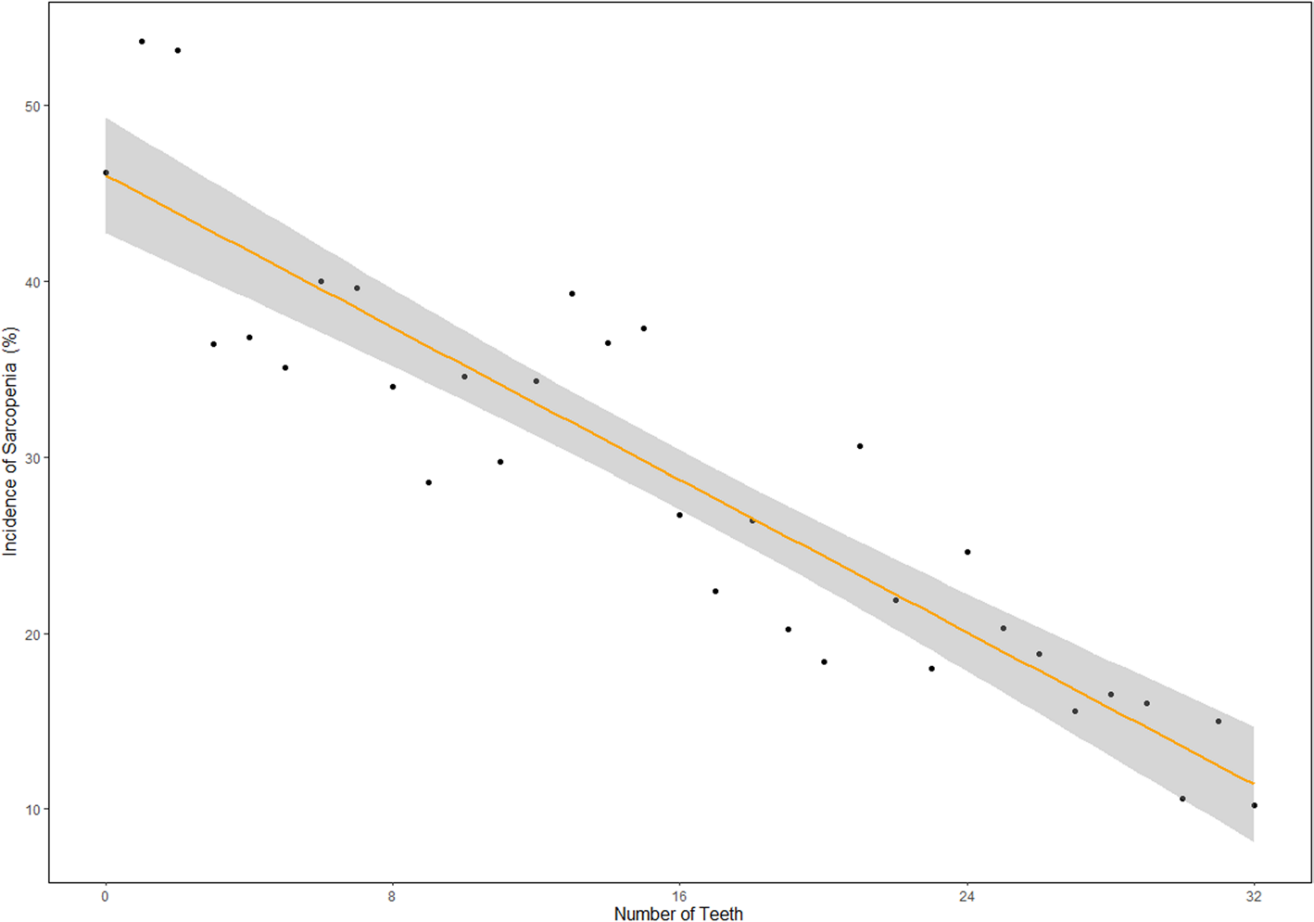
Distribution of sarcopenia prevalence with changes in the number of teeth

Overall, we enrolled 4149 participants (1498 male and 2651 female) aged 50 years old or older in the study. The mean age of the group was 62.4 ± 8.3 years. Table 1 shows descriptive characteristics of the participants according to sarcopenia assessment. 934 participants were found to be sarcopenia and the prevalence of sarcopenia was 22.5%. Subjects with sarcopenia tended to be older and with aging increasing, the prevalence of sarcopenia increased. It was observed that individuals in the sarcopenia group tended to be male, widowed, has a lower educational level, has smoking history, has cognitive decline and has false teeth (*p* < 0.001). And subjects with sarcopenia was more likely to live alone (*p* = 0.0018) and have chronic disease burden (*p* = 0.013). Besides, the prevalence of sarcopenia was higher in those subjects who were depressed (*p* = 0.0419) and has sleep disorders (*p* = 0.0332). Specifically, subjects with sarcopenia has a lower nutrition scores and with the nutrition status decline, the prevalence of sarcopenia was increasing (*p* < 0.001). Subjects in sarcopenia group has less number of teeth than non-sarcopenia group and with the teeth number decreasing, the higher of the prevalence of sarcopenia (*p* < 0.001). Specifically, distribution of sarcopenia prevalence with changes in the number of teeth could be seen in Fig. 2, indicated a linear correlation between number of teeth and sarcopenia.

**Table 1:** Sample characteristics stratified by sarcopenia status (*N* = 4149)

Characteristics	Total *N* = 4149	Non-sarcopenia ***N*** = 3215	Sarcopenia ***N*** = 934	***p*** value
**Age, Mean (SD)**	62.4 (8.3)	61.0 (7.6)	67.0 (8.8)	< 0.001
**Age group, N(%)**				< 0.001
**50–59**	1648 (39.7%)	1449 (87.9%)	199 (12.1%)	
**60–69**	1664 (40.1%)	1303 (78.3%)	361 (21.7%)	
**70–79**	709 (17.1%)	423 (59.7%)	286 (40.3%)	
**80+**	128 (3.1%)	40 (31.2%)	88 (68.8%)	
**Ethnic group, N(%)**				< 0.001
**Han**	1806 (43.5%)	1338 (74.1%)	468 (25.9%)	
**Non-Han**	2343 (56.5%)	1877 (80.1%)	466 (19.9%)	
**Gender, N(%)**				< 0.001
**Male**	1498 (36.1%)	1093 (73%)	405 (27%)	
**Female**	2651 (63.9%)	2122 (80%)	529 (20%)	
**Marriage status, N(%)**				< 0.001
**Without spouse**	97 (2.3%)	73 (75.3%)	24 (24.7%)	
**Have Spouse**	3487 (84%)	2762 (79.2%)	725 (20.8%)	
**Widowed**	565 (13.6%)	380 (67.3%)	185 (32.7%)	
**Educational level, N(%)**				< 0.001
**No formal education**	1253 (30.2%)	915 (73%)	338 (27%)	
**Elementary school**	1397 (33.7%)	1076 (77%)	321 (23%)	
**Middle school**	903 (21.8%)	741 (82.1%)	162 (17.9%)	
**High school and above**	596 (14.4%)	483 (81%)	113 (19%)	
**Smoking history, N(%)**				< 0.001
**No**	3428 (82.6%)	2718 (79.3%)	710 (20.7%)	
**Yes**	721 (17.4%)	497 (68.9%)	224 (31.1%)	
**Drinking alcohol history, N(%)**				0.7471
**No**	3093 (74.5%)	2401 (77.6%)	692 (22.4%)	
**Yes**	1056 (25.5%)	814 (77.1%)	242 (22.9%)	
**Drinking tea history, N(%)**				0.3532
**No**	2159 (52%)	1660 (76.9%)	499 (23.1%)	
**Yes**	1990 (48%)	1555 (78.1%)	435 (21.9%)	
**Number of teeth, Mean (SD)**	21.8 (9.3)	23.0 (8.5)	17.7 (10.5)	< 0.001
**Number of teeth group, N(%)**				< 0.001
**0–8**	550 (13.3%)	314 (57.1%)	236 (42.9%)	
**9–16**	431 (10.4%)	286 (66.4%)	145 (33.6%)	
**17–24**	924 (22.3%)	714 (77.3%)	210 (22.7%)	
**24–32**	2244 (54.1%)	1901 (84.7%)	343 (15.3%)	
**False teeth status, N(%)**				< 0.001
**No**	2686 (64.7%)	2157 (80.3%)	529 (19.7%)	
**Yes**	1463 (35.3%)	1058 (72.3%)	405 (27.7%)	
**Living alone status, N(%)**				0.0018
**No**	3951 (95.2%)	3080 (78%)	871 (22%)	
**Yes**	198 (4.8%)	135 (68.2%)	63 (31.8%)	
**Chronic disease status, N(%)**				0.013
**No chronic disease**	2423 (58.4%)	1873 (77.3%)	550 (22.7%)	
**One chronic disease**	954 (23%)	767 (80.4%)	187 (19.6%)	
**Two or more chronic diseases**	772 (18.6%)	575 (74.5%)	197 (25.5%)	
**Depressed status**				0.0419
**Non-depressed**	3371 (81.2%)	2634 (78.1%)	737 (21.9%)	
**Depressed**	778 (18.8%)	581 (74.7%)	197 (25.3%)	
**Sleep disorders, N(%)**				0.0332
**No**	2186 (52.7%)	1723 (78.8%)	463 (21.2%)	
**Yes**	1963 (47.3%)	1492 (76%)	471 (24%)	
**Anxiety status, N(%)**				0.4883
**No**	3323 (80.1%)	2567 (77.2%)	756 (22.8%)	
**Yes**	826 (19.9%)	648 (78.5%)	178 (21.5%)	
**Cognitive status, N(%)**				< 0.001
**No cognitive decline**	3570 (86%)	2828 (79.2%)	742 (20.8%)	
**Mild cognitive decline**	441 (10.6%)	317 (71.9%)	124 (28.1%)	
**Moderate to severe cognitive decline**	138 (3.3%)	70 (50.7%)	68 (49.3%)	
**MNA-SF score, Mean (SD)**	12.7 (1.5)	12.9 (1.3)	11.8 (1.7)	< 0.001
**Nutrition status, N(%)**				< 0.001
**Normal**	3330 (80.3%)	2767 (83.1%)	563 (16.9%)	
**Nutrition risk**	798 (19.2%)	442 (55.4%)	356 (44.6%)	
**Malnutrition**	21 (0.5%)	6 (28.6%)	15 (71.4%)	

*Note*. Means ± standard deviation was shown. Data are shown using % or mean (standard deviation). *p* values were calculated with chi-squared tests and student’s t tests for categorical and continuous variables, respectively

The results of multiple regression analysis in three different models was shown in Table 2. The covariates included gender, age, ethnic group, false teeth status, smoking, educational level, living alone status, marriage status, chronic diseases, depression, sleep disorders and cognitive function were adjusted in all the three models. In model 1, the results showed a significant relationship between number of teeth (β = − 0.327, 95% CI − 0.417 to − 0.237, *p* < 0.001) and sarcopenia. In Model 2, it showed that after adjusted nutrition scores, the association between number of teeth and sarcopenia was still significant (β = − 0.269, 95% CI − 0.364 to − 0.175, *p* < 0.001). In model 3, it showed a significant correlation between number of teeth (β = 0.157, 95% CI 0.107 to 0.207, p < 0.001) and nutrition scores.

**Table 2:** Regression results depicting the relationship between number of teeth and nutrition or sarcopenia

Outcome variable	Model 1: Sarcopenia			Model 2: Sarcopenia			Model 3: Nutrition (MNA-SF score)		
	***β***	***p*** value	95% CI	***β***	***p*** value	95% CI	***β***	***p*** value	95% CI
**Nutrition (MNA-SF score)**	–	–	–	− 0.473	< 0.001	− 0.532 to − 0.415	–	–	–
**Number of teeth**	− 0.327	< 0.001	− 0.417 to − 0.237	− 0.269	< 0.001	− 0.364 to − 0.175	0.157	< 0.001	0.107 to 0.207
**False teeth status: Yes**	− 0.195	0.051	−0.392 to 0	− 0.178	0.086	− 0.383 to 0.024	0.051	0.324	−0.051 to 0.153
**Gender: Female**	−0.303	0.004	−0.507 to − 0.098	−0.319	0.003	−0.53 to − 0.106	−0.011	0.84	−0.116 to 0.095
**Age: 60–69**	0.563	< 0.001	0.361 to 0.767	0.608	< 0.001	0.398 to 0.82	0.011	0.819	−0.084 to 0.107
**Age: 70–79**	1.164	< 0.001	0.919 to 1.411	1.231	< 0.001	0.975 to 1.49	− 0.074	0.274	− 0.208 to 0.059
**Age: 80+**	2.175	< 0.001	1.734 to 2.629	2.289	< 0.001	1.827 to 2.763	−0.183	0.162	−0.44 to 0.074
**Ethnic group: Non-Han**	−0.402	< 0.001	−0.566 to − 0.238	−0.431	< 0.001	− 0.602 to − 0.26	0.02	0.645	− 0.064 to 0.104
**Smoking history: Yes**	0.357	0.002	0.129 to 0.585	0.256	0.035	0.017 to 0.493	−0.257	< 0.001	− 0.383 to − 0.132
**Educational: Elementary school**	− 0.025	0.811	− 0.228 to 0.179	0.025	0.818	− 0.187 to 0.238	0.107	0.047	0.001 to 0.214
**Educational: Middle school**	−0.056	0.66	−0.303 to 0.191	− 0.006	0.962	−0.263 to 0.25	0.109	0.085	−0.015 to 0.232
**Educational: High school and above**	0.029	0.84	−0.252 to 0.306	0.101	0.494	−0.191 to 0.39	0.124	0.08	−0.015 to 0.263
**Marriage status: Have Spouse**	0.02	0.939	−0.489 to 0.563	−0.094	0.738	−0.628 to 0.475	− 0.214	0.123	− 0.485 to 0.058
**Marriage status: Widowed**	0.133	0.634	−0.401 to 0.695	−0.054	0.855	−0.615 to 0.537	− 0.361	0.013	− 0.647 to − 0.076
**Living alone status: Yes**	0.131	0.478	−0.236 to 0.487	0.004	0.983	−0.379 to 0.378	−0.235	0.02	−0.433 to − 0.037
**Chronic disease status: One**	− 0.319	0.002	− 0.521 to − 0.12	−0.271	0.011	−0.481 to − 0.063	0.14	0.006	0.041 to 0.239
**Chronic disease status: Two or more**	−0.288	0.007	−0.5 to − 0.079	−0.236	0.035	−0.457 to − 0.018	0.137	0.014	0.027 to 0.247
**Depressed status: Depressed**	0.129	0.21	−0.075 to 0.33	−0.03	0.784	−0.244 to 0.181	− 0.323	< 0.001	− 0.429 to − 0.218
**Sleep disorders: Yes**	0.055	0.509	− 0.109 to 0.219	− 0.019	0.825	− 0.191 to 0.152	−0.141	0.001	−0.225 to − 0.057
**Cognitive status: Mild**	0.228	0.076	−0.026 to 0.477	− 0.292	0.034	−0.566 to − 0.024	−1.094	< 0.001	−1.23 to − 0.959
**Cognitive status: Moderate to severe**	1.055	< 0.001	0.664 to 1.444	− 0.016	0.943	− 0.449 to 0.413	−2.447	< 0.001	−2.679 to − 2.216
**Constant**	− 0.434	0.217	−1.134 to 0.247	5.522	< 0.001	4.486 to 6.56	12.65	< 0.001	12.287 to 13.012

*Note*. Model 1: multiple linear regression analysis between sarcopenia and number of teeth; Model 2: multiple linear regression analysis between sarcopenia and number of teeth adjusted by MNA-SF score; Model 3: multiple linear regression analysis between MNA-SF score and number of teeth; All the models were adjusted by false teeth status, gender, age, ethnic group, life styles (smoking), educational, living alone status, marriage status, chronic diseases status, depression, sleep disorders and cognitive status

**Fig. 3:**
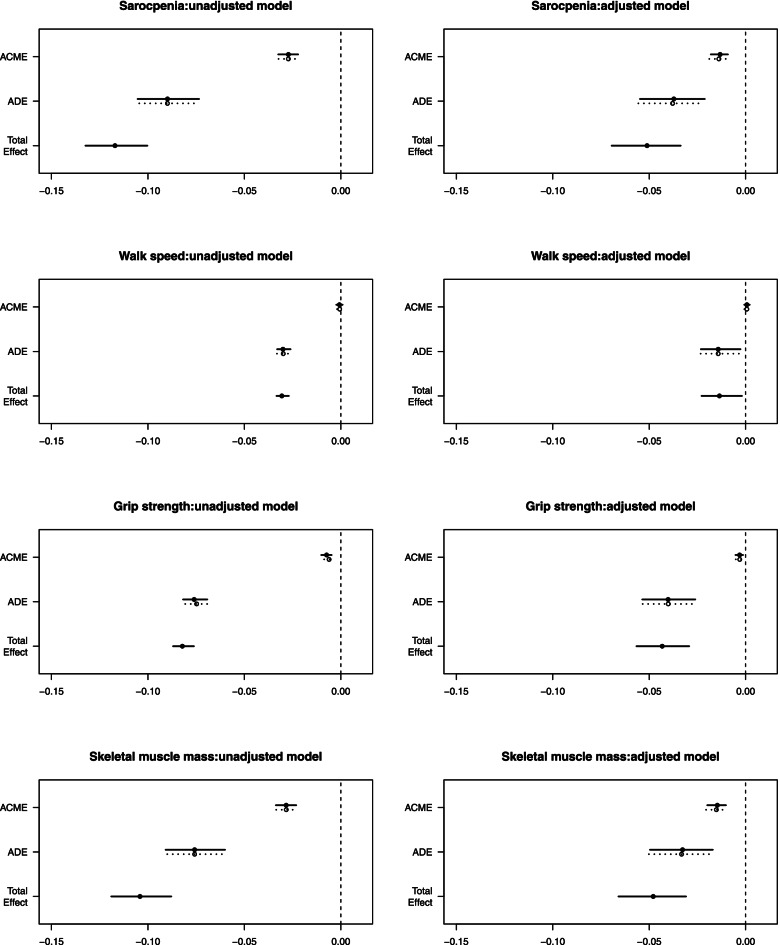
Mediation effects of nutrition in the relationship between number of teeth with sarcopenia and the three diagnostic components of sarcopenia (gait speed, grip strength, SMI) in an unadjusted model. Nutrition revealed significant relative indirect effects for number of teeth and sarcopenia (ACME = − 0.0272). Nutrition also revealed significant relative indirect effects for SMI (indirect effect estimate = − 0.0283) and grip strength (indirect effect estimate = − 0.0067)

The relative total, direct and indirect effects for the mediating role of nutrition on the relationship between number of teeth and sarcopenia in mediation models was shown in Table 3. Bootstrapping revealed significant relative indirect effects for sarcopenia (ACME = − 0.0272, 95% CI − 0.0324 to − 0.0222), indicating that nutrition mediated the association between number of teeth and sarcopenia. And after adjusting covariates including gender, age, ethnic group, smoking history, chronic disease status, and cognitive status, the mediation effect was still significant (ACME = − 0.0136, 95% CI − 0.0185 to − 0.0095). And the mediation effect with a proportion of mediation up to 23.24%. The mediation of nutrition in the relationship between number of teeth and the three components of sarcopenia was also analyzed. And this mediation effect was through impacting SMI (indirect effect estimate = − 0.0283, bootstrap 95% CI − 0.0336 to − 0.0232) and grip strength (indirect effect estimate = − 0.0067, bootstrap 95% CI − 0.0094 to − 0.0043). These mediation effects were also shown in Fig. 3. This suggested that nutrition status partially mediated the association between number of teeth and sarcopenia and this mediation effect was through impacting SMI and grip strength.

**Table 3:** Mediation models: relative total, direct and indirect effects for the mediating role of nutrition on the relationship between number of teeth and sarcopenia with the three components of sarcopenia (walking speed, grip strength, SMI)

Mediator Variable		Model1			Model 2		
		***β***	*p*-value	95% CI	***β***	p-value	95% CI
Sarcopenia	ACME	−0.0272	< 0.001	− 0.0324 to − 0.0222	−0.0136	< 0.001	− 0.0185 to − 0.0095
Sarcopenia	ADE	− 0.0899	< 0.001	− 0.1049 to − 0.0738	− 0.0376	< 0.001	− 0.0552 to − 0.0215
Sarcopenia	Total Effect	− 0.1171	< 0.001	− 0.1324 to − 0.1003	− 0.0511	< 0.001	− 0.0695 to − 0.0337
Sarcopenia	Prop.Mediated	0.2324	< 0.001	0.1863 to 0.2844	0.265	< 0.001	0.1816 to 0.3874
Walking speed	ACME	−0.0007	0.358	−0.0021 to 0.0008	0.0006	0.36	−0.0007 to 0.002
Walking speed	ADE	−0.0299	< 0.001	−0.0331 to − 0.026	−0.0142	0.022	−0.0233 to − 0.0027
Walking speed	Total Effect	−0.0306	< 0.001	− 0.0334 to − 0.027	−0.0136	0.032	−0.0229 to − 0.0019
Walking speed	Prop.Mediated	0.0211	0.358	−0.0277 to 0.0721	−0.0426	0.384	−0.3662 to 0.0785
Grip strength	ACME	−0.0067	< 0.001	−0.0094 to − 0.0043	−0.0031	< 0.001	− 0.0052 to − 0.0013
Grip strength	ADE	− 0.0754	< 0.001	− 0.0812 to − 0.0686	−0.0402	< 0.001	− 0.0535 to − 0.0261
Grip strength	Total Effect	− 0.0822	< 0.001	− 0.087 to − 0.0762	−0.0433	< 0.001	− 0.0566 to − 0.0293
Grip strength	Prop.Mediated	0.0816	< 0.001	0.0522 to 0.1168	0.0706	< 0.001	0.0291 to 0.1318
SMI	ACME	−0.0283	< 0.001	−0.0336 to − 0.0232	−0.0149	< 0.001	− 0.0202 to − 0.0104
SMI	ADE	− 0.0758	< 0.001	− 0.0904 to − 0.0602	−0.033	< 0.001	− 0.0499 to − 0.0172
SMI	Total Effect	− 0.1041	< 0.001	− 0.119 to − 0.0879	−0.0479	< 0.001	− 0.066 to − 0.0309
SMI	Prop.Mediated	0.2721	< 0.001	0.218 to 0.3352	0.3115	< 0.001	0.2134 to 0.4621

Note. ACME, average causal mediation effects (indirect effect); ADE, average direct effects; Prop. Mediated, the mediator variable explains the percentage of the association between cognitive and depressed. SMI, skeletal muscle mass

Model 1: None covariate was adjusted

Model 2: Adjusted by gender, age, ethnic group, smoking history, chronic disease status, and cognitive status

**Fig. 4:**
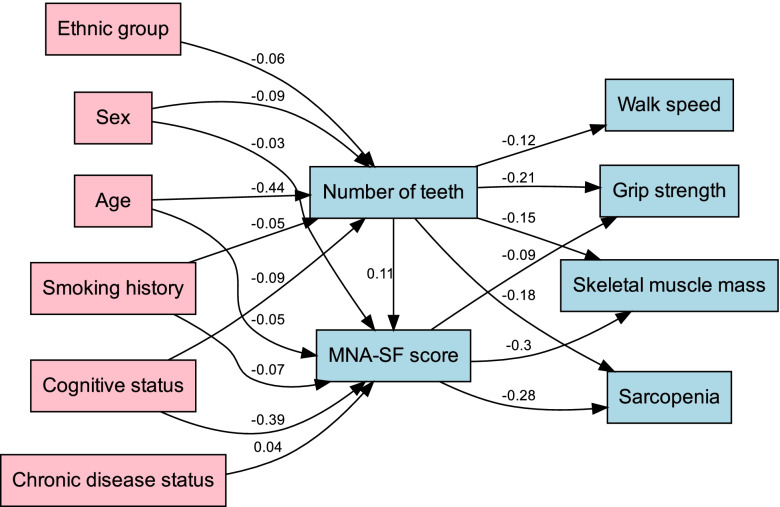
Path analysis of the nutrition’s mediation effects using the structural equation model (SEM) framework. SEM pathway analysis showed that the correlation between number of teeth and sarcopenia was negative (SEM co-efficient: − 0.18). The correlation between number of teeth and MNA-SF score was positive (SEM co-efficient: 0.11). The correlation between MNA-SF score and sarcopenia was negative (SEM coefficient: − 0.28)

We then performed pathway analysis using the structural equation model (SEM) framework. As shown in Fig. 4, SEM pathway analysis indicated that the association between number of teeth and sarcopenia was negative (SEM co-efficient: − 0.18). And the relationship between number of teeth and MNA-SF scores was positive (SEM co-efficient: 0.11). Besides, the correlation between MNA-SF scores and sarcopenia was negative (SEM coefficient: − 0.28). Moreover, other covariates including age, sex, ethnic groups, smoking history, and cognitive status showed negative estimate coefficients compared to number of teeth and MNA-SF scores. Furthermore, both number of teeth and MNA-SF score showed negative estimate coefficients compared to gait speed, grip strength and SMI. The *p* value was statistically significant in the entire pathway of the SEM structure model. These results confirmed the relationship between number of teeth, MNA-SF score and sarcopenia.

## Discussion

As far as we know, this was the first study to evaluate the mediating role of nutrition in the association between number of teeth and sarcopenia. Our study stated that nutrition could partially mediate the effects of teeth loss on sarcopenia in adults over 50 years old and the mediation effect was up to 26.5%. Therefore, better nutrition status could ameliorate negative effects of teeth loss on sarcopenia.

This study calculated the prevalence of sarcopenia using AWGS 2019 diagnostic criteria. The results demonstrated that the prevalence of sarcopenia was 22.5% (moderate sarcopenia: 9.0%; severe sarcopenia: 13.5%), a little higher than other studies. Ye C et al. analyzed data from China Health and Retirement Longitudinal Study which including 14,130 participants aged over 50 years old, showing the prevalence of sarcopenia was 19.8% (moderate sarcopenia: 11.9%; severe sarcopenia: 7.9%) [20]. Another meta-analysis summarized 9 studies and discovered that the prevalence of sarcopenia was 14% among 7656 participants aged over 50 years old living in community [21]. Considering that the participants in our study were recruited from rural areas, most of them had no formal education(*n* = 1253, 30.2%), which may have been the main reason for the high prevalence of sarcopenia [22]. Consisting with other studies, sarcopenia prevalence in our study was age-related (50-59ys, 12.1%; 60-69ys, 21.7%; 70-79ys, 40.3%; ≥80ys, 68.8%), indicating a dosage effect.

In our study, we found a significant negative association between number of teeth and sarcopenia, after adjusting false teeth status, gender, age, ethnic group, life styles (smoking), educational level, living alone status, marriage status, chronic diseases status, depression, sleep disorders and cognitive status. This was consistent with other studies. One study found elderly patients with less than 20 number of teeth were more likely to have diagnosed sarcopenia (OR 5.79, 95%CI 3.90–8.59) or severe sarcopenia (OR 7.32, 95%CI 4.28–12.52) in adjusted model compared with patients having more than 20 number of teeth [8]. Another study also found that number of teeth was associated with low handgrip strength (OR = 0.961; 95% CI, 0.932–0.992) and possible sarcopenia (OR = 0.949; 95% CI, 0.907–0.992) [7]. Besides, the remaining 0–9 teeth was found to be associated with low grip strength compared to high grip strength in men (OR 1.39, 95% CI 1.03–1.88) [23]. The underling mechanism was as following. Firstly, less number of teeth led to poor chewing ability and dietary intake, thus leading to malnutrition which was a risk factor in sarcopenia [10]. Secondly, elderly who have less number of teeth would less likely to eat meat, nut and other hard or raw food. And this kind of food contain protein, calcium and vitamins, playing an important role in maintaining muscle mass [24]. Thirdly, the number of missing teeth could reflect the cumulative oral inflammatory status which was also a factor in the mechanism of sarcopenia [25]. This meant that number of teeth was an important factor related with sarcopenia with a dosage effect. And sarcopenia prevalence in our study ranging from 15.3% in those individuals with 24–32 teeth to 42.9% in those individuals with 0–8 teeth.

Specifically, we found a partial mediation effect of nutrition in the relationship between number of teeth and sarcopenia. The mediation model and SEM framework pathway analyses showed that the mediation effect of nutrition was high up to 26.5% in adjusted model. There are several plausible explanations for our findings. Firstly, there is a close relationship between number of teeth, nutrition status, and sarcopenia. A recent study has confirmed that improving nutrition and oral health may be an effective way to reduce or delay the occurrence of sarcopenia [8]. Sarcopenia can also impact swallowing related muscle. Secondly, individuals who had less teeth increased the risk of being malnutrition [26]. Elderly who has few or no natural teeth would select a diet that they can chew in comfort. Such diets are low in fruits and vegetables intake with associated reduction in dietary nutrition intake [27]. Conversely, malnourished older adults are likely to have poorer oral function, leading to more teeth loss [28]. Thirdly, oral health can be associated with balance and exercise through neuronal mechanism. And some research had found that dental occlusion influenced postural stability [29]. Nutrition therapy is also essential to improve muscle mass and function in patients with sarcopenia [30]. Therefore, malnutrition promotes teeth loss, which further increases the risk of sarcopenia in the elderly. Use false teeth is important to promote early intervention in nutrition and sarcopenia.

Among the 3 components of sarcopenia (speed, grip strength and SMI), we found the mediation role of nutrition was through impacting grip strength and SMI, not gait speed. This was consistent with previous studies. Kim et al. found that the participants with NRT ≥ 20 had more SMI than those with NRT < 20 in both sexes. And SMI was correlated with number of teeth in men (r = 0.018, *p* < 0.001) and in women (r = − 0.007, p < 0.001) [31]. Another study found that hopeless teeth and less posterior occlusion was related to a greater risk of low handgrip strength [32]. Furthermore, lack of posterior occlusal support at baseline was found to be independently predicted the incidence of declined gait speed over 3 years [33]. In a word, a longitudinal study was required to confirm our findings.

There are some limitations in this study. Firstly, most participants enrolled in our study were relatively younger and healthy. Secondly, we only analyzed baseline data of the WCHAT study. Thirdly, we did not assess oral health including chewing ability, articulatory oral motor skill, tongue pressure, and swallowing. All of these were potential source of bias. A longitudinal study was required to establish the relationship between number of teeth, nutrition and sarcopenia.

## Conclusions

In conclusion, our study demonstrated that number of teeth is negatively associated with sarcopenia and the relationship was partially mediated by nutrition. Our results supported the need for early identification and interventions for malnutrition in at-risk older adults with teeth loss to prevent the downstream cascade of sarcopenia.

## Data Availability

The data-set generated and analyzed during the current study will be available from the corresponding author on a reasonable request.

## Abbreviations

WCHAT: West China Health and Aging Trend study
GDS-15: 15-item Geriatric Depression Scale
SPMSQ: Short Portable Mental Status Questionnaire
PSQI: Pittsburgh sleep quality index
SEM: Structural equation model
MNA-SF: Mini Nutrition Assessment-Short Form scale
ACME: Average causal mediation effects (indirect effect)
ADE: Average direct effects
